# Mechanism of dentin hardness measurements using a dentin hardness measuring device with a light-emitting diode

**DOI:** 10.1117/1.JBO.29.2.025002

**Published:** 2024-02-22

**Authors:** Sota Kondo, Yutaka Tomioka, Naohiro Fujimoto, Atsushi Mine, Satoshi Yamaguchi, Saeko Okumura, Hiroaki Tanimoto, Kenzo Yasuo, Kazushi Yoshikawa, Kazuyo Yamamoto, Hisanao Hazama

**Affiliations:** a Osaka University, Graduate School of Engineering, Osaka, Japan; b National Cancer Center Hospital East, Department for the Promotion of Medical Device Innovation, Chiba, Japan; c Osaka University Graduate School of Dentistry, Department of Fixed Prosthodontics and Orofacial Function, Osaka, Japan; d Osaka University Graduate School of Dentistry, Department of Dental Biomaterials, Osaka, Japan; e Osaka Dental University, Department of Operative Dentistry, Osaka, Japan

**Keywords:** dentin, hardness, light-emitting diode, root caries, demineralization, diagnosis

## Abstract

**Significance:**

Managing caries is imperative in a rapidly aging society. Current diagnoses use qualitative indices. However, a quantitative evaluation of hardness in a clinical setting may lead to more accurate diagnoses. Previously, hardness meter using indenter with light for tooth monitoring (HAMILTOM) was developed to quantitatively measure tooth hardness. Herein, the physical interpretation of dentin hardness measured using HAMILTOM and the dentin hardness measurement mechanism are discussed.

**Aim:**

This study evaluates the mechanism of dentin hardness measurements using HAMILTOM physically and compare the invasiveness to dentin by HAMILTOM with those using a dental probe for palpation.

**Approach:**

Eleven bovine dentin samples were used to create caries models. HAMILTOM measured the dark areas, and its indentations were observed using scanning electron microscopy. Also, its invasiveness was evaluated by comparing the results with those from dental probe palpation.

**Results:**

The indentation areas were smaller than the dark areas in HAMILTOM, which may be due to exuded water from the dentin sample and the elastic recovery of dentin sample. Additionally, the dental probe indentation was deeper than the HAMILTOM indentations.

**Conclusions:**

The results demonstrate that the indentation areas were smaller than the dark areas measured by HAMILTOM, which might contain the influence of exuded water and the deformation of dentin sample. Also, HAMILTOM is less invasive than dental probe palpation. In the future, HAMILTOM may become a standard hardness measuring method to diagnose root caries.

## 1 Introduction

The world’s population is not only increasing but it is also aging. According to the World Health Organization (WHO), one in six people will be 60 years or older by 2030, and the world’s population over 60 years of age will reach 2.1 billion by 2050.^1^ As life expectancy of the aging population increases, people retain their teeth for longer,^2^ increasing the risk of developing caries. Especially, root caries is a dental disease affecting more than one-third of the geriatric population.^3^

Root caries is common on exposed root surfaces or margins of the cementoenamel junction.^4^ It is defined as a cavitation below the cementoenamel junction, including both cementum and underlying dentin but not the adjacent enamel. The cavitation is usually discolored, softened, and ill-defined.^5^ Enamel is stronger and more acid-resistant than any other dental tissue since it contains about 90% minerals.^6^ Cementum and dentin are composed of about 45–50% and 70% inorganic materials, respectively.^7^^,^^8^ Additionally, the higher content of magnesium and carbonate makes cementum and dentin more soluble than enamel.^9^ Compared to enamel, cementum and dentin on a root surface are more susceptible to caries progression and is more likely to cause tooth loss. Root caries begins with demineralization of cementum after exposure of the root surface due to gingival recession, and spreads to the dentin. Cementum is a tissue similar to dentin and has lower acid resistance than enamel, making it more susceptible to caries. Caries progresses against the dentin by collapsing and shedding the cementum, exposing a wide area of dentin. The thickness of the cementum in cervical area is thin, approximately 20 to 50  μm, and most root caries identified by inspection is dentin caries.^10^ Therefore, this study focused on dentin because it is considered important to evaluate the progression of root caries to dentin.

The treatment of root caries is much more difficult than that of the crown because the cutting of root surface caries could break the tooth, and the proximity to the gingiva could make cement filling difficult.^10^ Therefore, it is important to assess the progression of root caries through diagnosis to prevent severe root caries. Root caries is diagnosed by inspection and palpation, using indices, such as color, surface texture, and lesion hardness.^10^^,^^11^ Obvious caries lesions may be seen on a simple visual clinical examination.^12^ Adjunctive information or evidence of enamel roughness and softening of dentin is provided by the palpation method using an explorer or dental probe.^13^ In the WHO method, lesions are classified into two stages (leathery, soft) by palpation with a community periodontal index (CPI) dental probe.^14^ In addition, the International Caries Detection and Assessment System, which is an international caries diagnosis and assessment system, has proposed a clinical classification of the depth of the actual lesion determined using the tip of the CPI dental probe for the pathology of root caries.^15^ However, the initial caries does not show a clear color change.^16^

Because palpation is performed using a dental probe, the inspection for root caries diagnosis is qualitative. As caries progresses, dentin becomes softer. However, diagnosis using a dental probe varies by dentist because it depends on the dentist’s sensitivity to pressure changes when inserting and removing a dental probe.^10^ In fact, the kappa statistics for the inspection and palpation for root caries are as low as 30% to 51%,^17^ suggesting that the probability of agreement of inspection and palpation results of root caries among different dentists is low. Consequently, a more quantitative diagnosis is required.

An indirect method of tracking the changes in the mineral content of dentin is hardness testing.^18^^,^^19^ However, an objective and quantitative method to evaluate the activity and progression of root caries in a clinical setting has yet to be established. A quantitative evaluation of hardness of *in vivo* teeth in the clinical setting may accurately evaluate the activity and progress of root caries, which may increase early detection of initial root caries and reduce the risk of becoming severe.

Typically, Vickers or Knoop hardness is used as an indicator of tooth hardness.^20^ Both tests evaluate hardness by measuring the indentation size after pressing and removing an indenter of a quadrangular pyramid with a constant load on the target object. A drawback of these methods is that the measurement requires a dry sample because samples with a large elastic deformation like a tooth with caries does not maintain the indentation. Therefore, hardness measurements cannot be performed on an *in vivo* tooth. The tooth must be extracted prior to evaluation.

Another technique to quantify the hardness of teeth in research is Cariotester. Cariotester is registered as a medical device in Japan. In this technique, paint is applied to the tip of a metal indenter and the length of the part where the paint disappears after contacting the tooth is measured.^21^ Because the measurement procedure is complicated and requires a microscope for observations, this technique is difficult to utilize in clinical settings.

Here, hardness meter using indenter with light for tooth monitoring (HAMILTOM) was developed as a technology that could easily and quantitatively evaluate tooth hardness using a light-emitting diode (LED). HAMILTOM consists of a system composed of an LED, a film diffuser, a beam splitter, a transparent conical indenter, a lens, and a complementary metal-oxide-semiconductor (CMOS) camera in the lens barrel, and a capacitive load sensor for controlling the load on the indenter and in-house control boards are placed in a handpiece-type polycarbonate housing. The measurement principle of HAMILTOM is based on a total internal reflection using the differences in the refractive indexes of air, the indenter, and dentin.^22^ HAMILTOM quantifies the hardness of dentin from the contact projection area (dark area) between the indenter and dentin when the indenter is pressed into the dentin. A previous proof-of-principle study confirmed the strong correlation between the Vickers hardness and HAMILTOM measurements. The coefficient of determination between the Vickers hardness converted from the dark areas measured by HAMILTOM and the Vickers hardness measured by the Vickers hardness tester was 0.99,^22^ suggesting that HAMILTOM has potential to quantitatively evaluate the hardness of *in vivo* teeth. However, the measurement mechanism of HAMILTOM remains unclear and the definition of “hardness” measured using HAMILTOM should be clarified. In particular, it was emphasized that the dark areas were not the size of the indentation created on the dentin and the influence of the water exuded by the contact of the indenter was significant, and it was considered that the indentation did not remain due to the elastic recovery of the dentin after removing the indenter.^22^ However, it was the hypothesis and needed to be verified. Therefore, the dentin hardness measured using HAMILTOM is necessary to be interpreted physically. In addition, a safety evaluation regarding invasiveness is required in order to use the device in clinical practice. The invasiveness of palpation is a concern. When a dental probe is used under load, iatrogenic damage of the enamel surface may occur, promoting caries progression.^12^^,^^23^

This study physically interprets the dentin hardness measured using HAMILTOM and considers its measurement mechanism. Additionally, the invasiveness to dentin during a hardness measurement using HAMILTOM is evaluated by comparing to the invasiveness of palpation by dental probe.

## 2 Materials and Methods

### 2.1 Sample Preparation

The samples were 11 bovine sound dentins. Bovine dentin samples were used for two reasons. First, samples were easily collected since bovine dentin samples are larger than human ones. Second, samples with uniform sizes were obtained, allowing multiple points to be measured in each sample.

Caries simulated models with various hardness were prepared by artificially demineralizing sound dentin samples for a time of 0.25, 0.5, 0.75, 1, 2, 4, 6, 12, or 24 h. Then HAMILTOM measured the dark areas in these models. Additionally, the indentations made by HAMILTOM or a dental probe were observed using a scanning electron microscope (SEM, JCM-5700, JEOL, Japan). This study was performed after obtaining approval from the Animal Care and Use Committee, Osaka Dental University (Approval No.:19-12001).

The samples were prepared by cutting, embedding in epoxy resin, curing for at least 24 h, and mirror polishing the surface.^22^ First, the extracted bovine tooth was cut perpendicular to the running direction of the dentinal tubules, and a manicure was applied to the back and all sides of the dentin samples prior to embedding to protect the dentinal tubules from the epoxy resin solution. Second, the dentin samples were embedded in an epoxy resin (Crystal Resin, NISSIN RESIN, Japan) using silicone molds. The samples were cured for at least 24 h, and then they were removed from the molds. Then, the sample surfaces were mirror polished under water injection with waterproof abrasive papers #400, 800, and 2000 (Kohnan, Japan) and a lapping film #8000 (LF1D, Thorlabs, United States) to create a horizontal hardness measurement surface. The sample tilt was adjusted to 1 deg or less during polishing for the sample orientation of 90 deg to the indenter. In addition, ultrasonic cleaning was performed for 2 min using a desktop ultrasonic cleaner (B2210, Branson Ultrasonic, United States) to remove the resin pieces and the inorganic components of dentin generated by polishing from the dentinal tubules.

To realize caries models with different hardness, the samples were immersed in a lactic acid solution for artificial demineralization.^24^^,^^25^ Lactic acid (20006-75, Nacalai Tesque, Japan) and distilled water were mixed in a beaker to prepare 1 L of 0.1 M demineralization solution. The beaker was placed in a constant temperature water bath (TM-3A, AS ONE, Japan) to hold the solution temperature at 37°C. The solution was agitated with a stirrer (HE-16GA, KPI, Japan) at a rotation speed of 600 rpm to maintain a pH of 2. Next, each dentin sample was immersed in the solution for a predetermined time of 0.25, 0.5, 0.75, 1, 2, 4, 6, 12, or 24 h. After the demineralization, the samples were rinsed with tap water and stored in saline (Otsuka Pharmaceutical, Japan) at 4°C.

Nine samples were used to create a caries model with a different demineralization time, and the remaining two were stored as sound dentin samples without demineralization. Here, the dark area was measured using HAMILTOM. Additionally, the indentation area by HAMILTOM or the dental probe was measured using the SEM.

The validity of the artificial demineralization by the acid etching was evaluated by measuring the actual mineral loss of the dentin samples used in this study with an energy dispersive X-ray spectrometer (EDS, JED-2300, JEOL, Japan) attached to the SEM used in this study. After the SEM observation of the indentations on the dentin samples, the dentin samples with different demineralization times were cut perpendicular to the surface in half so that the cross-section of the dentin sample was exposed. Next, the surfaces of the cross-section were mirror polished under water injection using waterproof abrasive papers #80, 220, 500, 1000, and 2000 (S31TO, IMT, Japan) and #4000 (S31HM, IMT, Japan). Then, the polished surfaces of the dentin samples were coated with about 4-nm-thick gold using an ion-sputtering system (E-1010, Hitachi, Japan) for 30 s at a 15-mA current to prevent overcharge of the sample. The Ca content distribution in the cross-section of each dentin sample was visualized by element mapping using the EDS, and the Ca content of the dentin sample surface where the dark areas were measured with HAMILTOM was also measured at three points in each dentin sample. The accelerating voltage and the magnification were set to 15 kV and 300×, respectively.

### 2.2 Dentin Hardness Measuring Device Using a Light-Emitting Diode

#### 2.2.1 Device setup and measurement principle

**Fig. 1:**
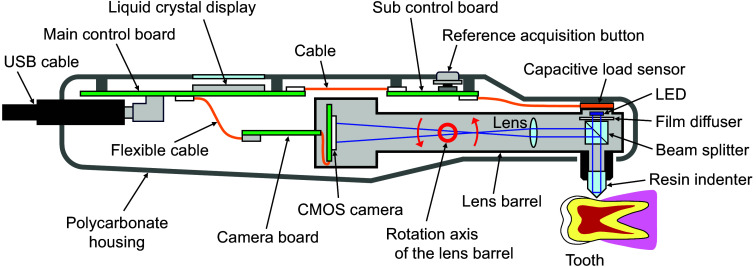
Schematic illustration of the HAMILTOM handpiece. Image of the resin indenter tip is projected onto the CMOS camera with a 1.5-fold magnification using LED light. By applying a load to the indenter tip, the lens barrel rotates around the rotation axis, and the lens barrel and the housing apply a load to the load sensor. Then CMOS acquires an image of the indenter when the load reaches a predetermined value.

Figure 1 schematically illustrates HAMILTOM. HAMILTOM measures dentin hardness from the dark area, which is the contact area between the indenter and the dentin. HAMILTOM includes a system composed of an LED with a 455-nm center wavelength, a film diffuser (#17-682, Edmund Optics, USA), a beam splitter (#47-007, Edmund Optics), a transparent conical indenter with a 90 deg apex angle, a lens with a 30-mm focal length (#45-134, Edmund Optics), and a CMOS camera (ID1MB-MDL-U, iDule, Japan) in the lens barrel. The indenter is composed of a photocuring resin and has a refractive index of 1.54, which is close to the refractive index of dentin (1.54).^26^ The LED light passing through the film diffuser is incident on the indenter. A beam splitter reflects light toward the camera, and the image of the indenter tip is projected on the CMOS camera using a lens with about 1.5-fold magnification. A capacitive load sensor with an 8-mm diameter and 0.3-mm thickness (SingleTact S8-1N, Pressure Profile Systems, United States) and in-house control boards are placed in a handpiece-type polycarbonate housing to measure a load to the tip of the indenter continuously and measure the dark area at a constant contact load during the hardness measurement with HAMILTOM. The reference acquisition button is used to obtain a reference image before the indenter is brought into contact with the sample. As for the principle of load measurement, by applying a load to the tip of indenter contacting to the dentin sample, the lens barrel rotates around the rotation axis. Then the load is applied to the load sensor by the motion of the lens barrel toward the housing. The applied load to the load sensor is continuously monitored on a tablet PC (Surface Go 2, Microsoft, United States) through the control boards with a USB interface. Then when the load measured by the load sensor reaches a predetermined value, the CMOS camera automatically acquires an image of the indenter using in-house software.

The measurement principle of HAMILTOM is based on a total internal reflection using the differences in the refractive indexes of air, the indenter, and dentin.^22^ When the indenter is not in contact with the dentin, the indenter appears bright because a total internal reflection occurs at the boundary between the indenter and the air. However, a total internal reflection does not occur when the indenter comes in contact with the dentin because the refractive index of the indenter and dentin are close. For a constant contact load, a softer dentin should yield a larger dark area than a harder dentin. Dentin becomes softer as caries progresses. Hence, the dark area may indicate the degree of caries progression because the dark area of the caries dentin should increase compared to the sound dentin.

#### 2.2.2 Calculation of the dark area and Vickers hardness

**Fig. 2:**
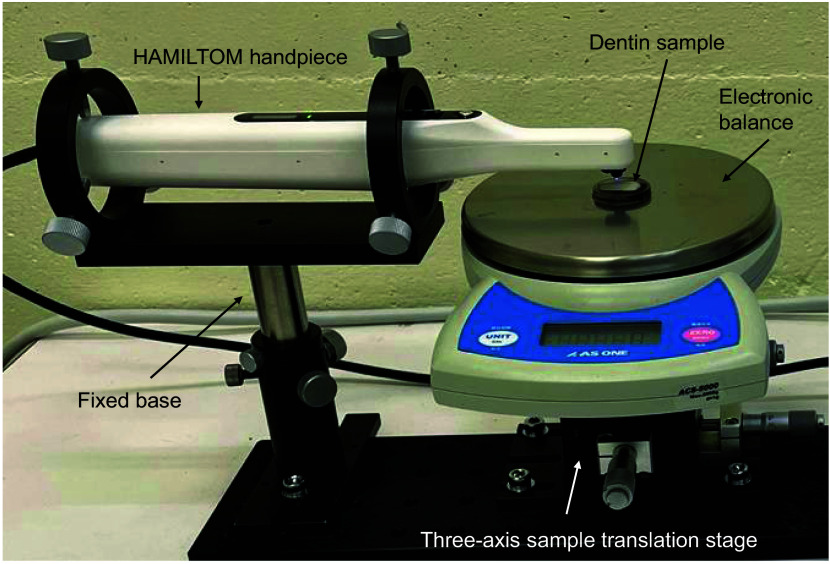
Image of the experimental system to measure dark areas by HAMILTOM. HAMILTOM is fixed at the base, and the dentin sample is placed directly below the indenter. The stage is slowly raised vertically. When the load reaches 0.49 N, an image is acquired with the CMOS camera. To verify the accuracy of the load measured by the sensor, the load is also measured by an electronic balance.

The dark areas were measured using HAMILTOM for the dentin sample at each demineralization time. Prior to each measurement, the dentin sample was moistened by immersing in saline for at least 24 h. Figure 2 shows a photograph of the experimental setup to measure the dark areas using HAMILTOM under a stable condition. HAMILTOM was fixed so that the indenter tip was oriented vertically downward. The excess surface moisture was blown off with air, and the dentin sample was placed on the stage directly below the indenter. The threshold of the load sensor when calculating the dark area was set to 0.10, 0.29, 0.49, 0.98, 1.47, 1.96, or 2.45 N. The stage was manually raised in the vertical direction. The CMOS camera acquired an image once the load reached the specified values. The load of 0.49 N was also measured by an electronic balance (ACS-5000, AS ONE, Japan) to verify the load accuracy. The accuracy of the electronic balance was confirmed before and after the experiments using a standard weight set (3-9951-04, AS ONE). Before the experiment, a coefficient, which was multiplied with the measured value of the load sensor, was adjusted to match the load measured with the electronic balance. After setting the dentin sample on the electronic balance, the electronic balance was reset to zero because the weight of each sample differed slightly. Images of the dark area were acquired at three different positions for each dentin sample. To make it easier to find the indentation points after the first measurement, the measurement interval was standardized to 500  μm using the stage. After each measurement, a lens cleaning paper (EK1546027S, Tiffen, United States) with ethanol was used to remove the water and deposits on the indenter surface.

To calculate the dark area, a reference image was obtained before the indenter was brought into contact with the sample. Once the reference image was acquired, the load measured with the load sensor was reset to zero to cancel the long-term drift of the sensor. Next, an image was acquired after the indenter was brought into contact with the sample. Then the reference was subtracted. A binarized image was created using a threshold of 50% of the maximum brightness of the subtracted image. In the binarized image, the contact area appeared as bright pixels, and the dark area was calculated from the number of bright pixels in the binarized image. The Vickers hardness of the dentin samples was calculated from the dark area measured at the load of 0.49 N using the following calibration curve obtained in our previous research^22^

$$H_{\text{V}}=13.86A^{-0.21},$$ (1)

where $H_{\text{V}}$ (HV) is the Vickers hardness, and A $\left({\mathrm{mm}}^{2}\right)$ is the dark area.

### 2.3 Evaluation

For each dentin sample with a different demineralization time, the dark areas were measured using HAMILTOM with the 0.49-N load at three random positions. Then the indentations made by HAMILTOM were observed using the SEM to verify the hypothesis that the dark areas were not the size of the indentation created on the dentin. Prior to the observations, the dentin samples were dried, and the surfaces of the dentin samples were coated with about 4-nm-thick gold using the ion-sputtering system for 30 s at the 15-mA current to prevent overcharge of the sample. Additionally, the indentation depths were measured using a confocal laser-scanning microscope (OPTELICS HYBRID, Lasertec, Japan).

For each demineralization time, the measured dark areas and the indentation areas in the SEM images were compared. The indentation area was calculated as an oval shape by measuring the long and short outer diameters of the indentation from the SEM images. Before the SEM observation, the scale bar accuracy was confirmed using a nickel mesh (EM-fine grid F-400 mesh, Nisshin-EM, Japan). The size of one mesh square was 63.5  μm, which was the same as that measured by the SEM scale bar.

Indentations of HAMILTOM with various loads of 0.10 to 2.45 N or a dental probe (AD1 #5, YDM, Japan) based on palpation by a dentist belonging to Osaka Dental University were created at three random positions for each sound dentin sample. To moisten the dentin sample, it was immersed in saline for at least 24 h. Then the excess surface moisture was blown off with air prior to indentation creation. The dentist applied the same load as a clinical setting to create indentations on the dentin sample surface using a dental probe. In actual clinical palpation, the probe is contacted with dentin and then slid a few millimeters to check dentin hardness. Therefore, the same probing operation with a long scratch was used in this study. The indentations were observed using SEM after the dentin sample was dried, and the depths were measured using the confocal laser-scanning microscope. Finally, the invasiveness of HAMILTOM was compared to that of palpation using a dental probe.

## 3 Results

**Fig. 3:**
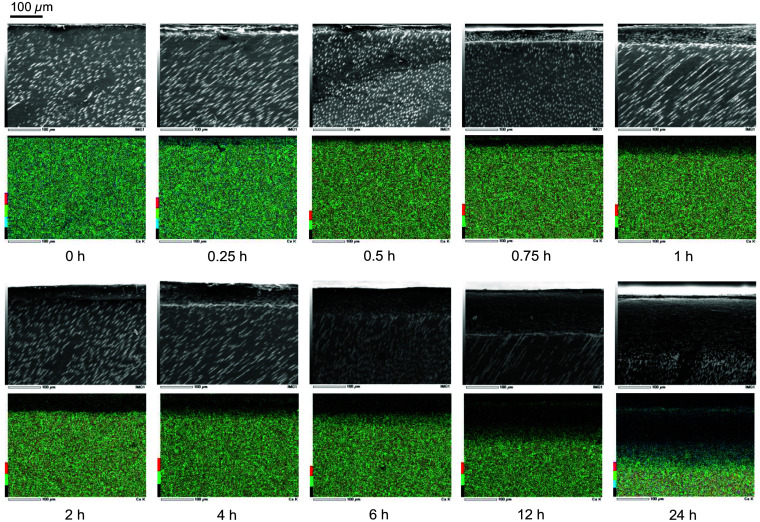
SEM images and mapping of Ca content in the cross-section of each dentin sample with the different demineralization times (0.25, 0.5, 0.75, 1, 2, 4, 6, 12, or 24 h) measured with the EDS attached to the SEM used in this study. The top side of the images illustrates the dentin sample surface on which the indentations were created in this study.

**Fig. 4:**
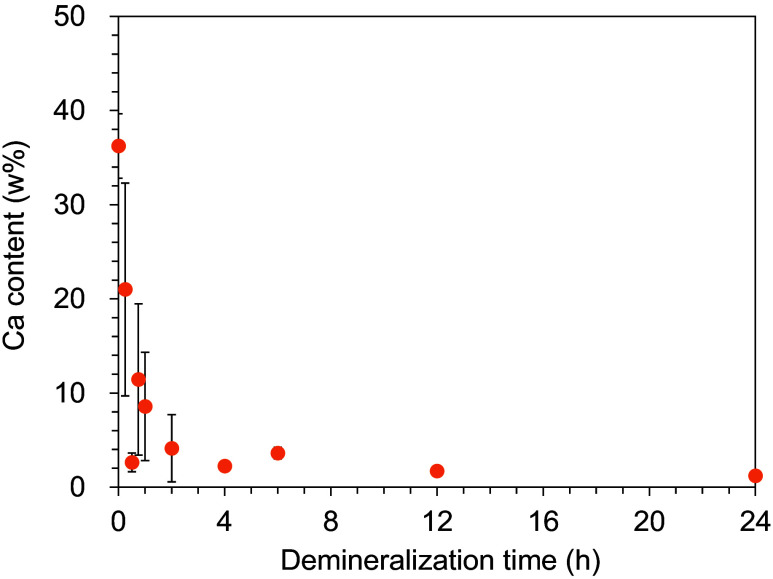
Ca content of the dentin sample surface, where the dark areas were measured with HAMILTOM, at three points in each dentin sample with the different demineralization times (0.25, 0.5, 0.75, 1, 2, 4, 6, 12, or 24 h) measured with the EDS attached to the SEM used in this study.

Figure 3 shows the SEM images and the mapping of Ca content in the cross-section of each dentin sample with the different demineralization time measured with the EDS attached to the SEM used in this study. The top side of the images illustrates the dentin sample surface on which the indentations were created in this study. A gradual decrease in the Ca content was observed from the dentin sample surface with increasing demineralization time. Figure 4 shows the Ca content of the dentin sample surface, where the dark areas were measured with HAMILTOM, at three points in each dentin sample with the different demineralization time measured with the EDS attached to the SEM used in this study. A clear difference in Ca content was observed between sound and demineralized dentin samples. The variation in Ca content with 0.25 to 2 h of demineralization time was large, but as the demineralization time increased, the Ca content on the dentin sample surface decreased, ranging from 1% to 2% at the demineralization time of 12 h or longer. From the observation of the change of Ca content in each dentin sample with the different demineralization time, the validity of the artificial demineralization by the acid etching in this study was confirmed.

**Fig. 5:**
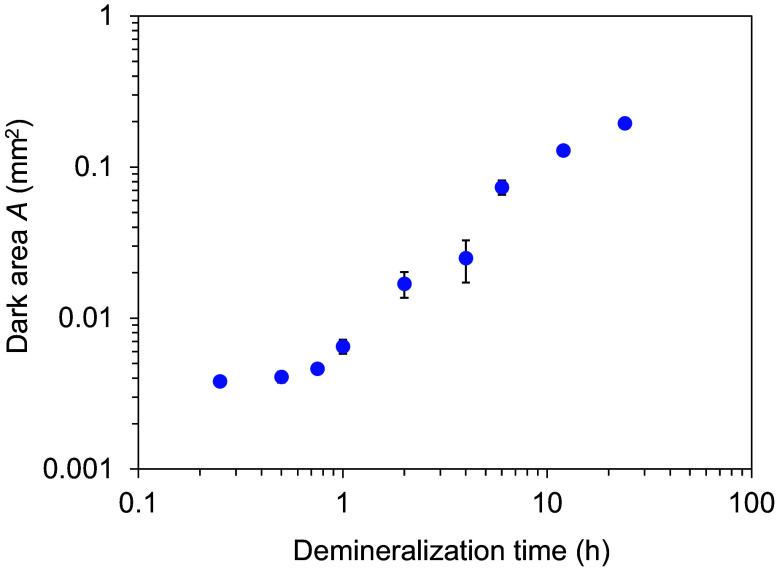
Changes in the dark area with different demineralization times (0.25, 0.5, 0.75, 1, 2, 4, 6, 12, or 24 h). Graph shows the mean and standard deviation of three measurements.

**Fig. 6:**
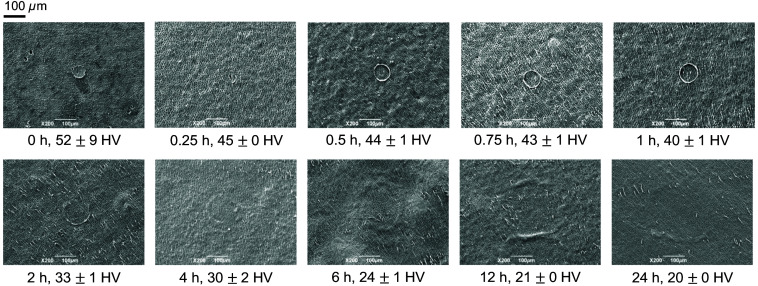
SEM images of indentations on dentin samples with different demineralization time (0, 0.25, 0.5, 0.75, 1, 2, 4, 6, 12, or 24 h) and different Vickers hardness (52±9, 45±0, 44±1, 43±1, 40±1, 33±1, 30±2, 24±1, 21±0, or 20±0  HV) after hardness measurements with HAMILTOM.

Figure 5 shows the changes in the dark area measured using HAMILTOM with the load of 0.49 N with different demineralization time. The load values measured by the electronic balance were 0.45 to 0.53 N when the dark areas were measured using HAMILTOM with the load of 0.49 N. The size of the dark area increased with demineralization time. Table 1 shows the dark area and the Vickers hardness of the dentin samples calculated with the calibration curve expressed as Eq. (1).^22^ The Vickers hardness was in a range from 20 to 52 with the demineralization time from 0 to 24 h. Figure 6 shows SEM images of the indentation at each demineralization time and Vickers hardness left on the dentin sample surface after the measurement of dark areas using HAMILTOM. HAMILTOM generated an indentation for all demineralization times. The indentation area increased with demineralization time, but the indentation shape was indistinct for demineralization times longer than 4 h. In the sound dentin sample without demineralization treatment, dentinal tubules in the indentation were closed, whereas the dentinal tubules in the indentation in demineralized dentin samples were open.

**Table 1:** Dark area and the Vickers hardness of the dentin samples calculated with the calibration curve expressed as Eq. (1).^22^ Mean and standard deviation (SD) of three measurements are shown.

		Demineralization time (h)									
		0	0.25	0.5	0.75	1	2	4	6	12	24
Dark area (${\mathrm{mm}}^{2}$)	Mean	0.0029	0.0038	0.0041	0.0046	0.0065	0.0169	0.0250	0.0736	0.1290	0.1950
	SD	0.0024	0.0002	0.0003	0.0003	0.0007	0.0033	0.0078	0.0079	0.0068	0.0080
Vickers hardness (HV)	Mean	52	45	44	43	40	33	30	24	21	20
	SD	9	0	1	1	1	1	2	1	0	0

**Fig. 7:**
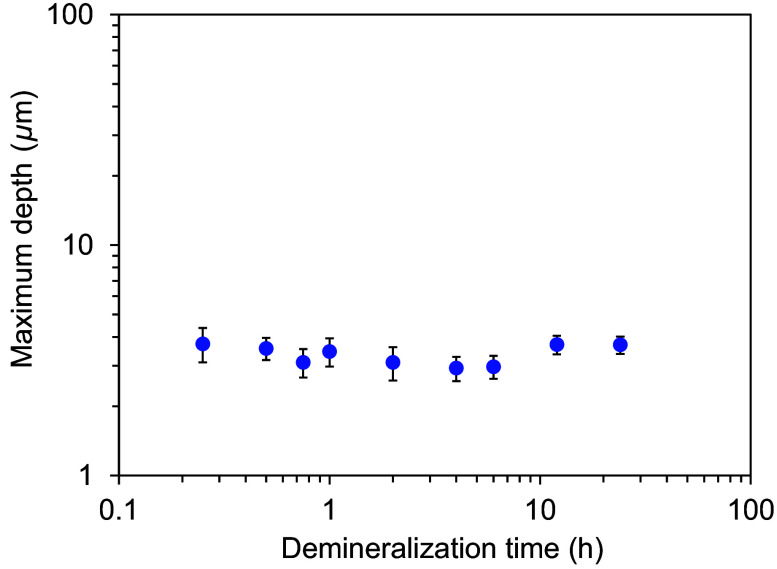
Changes in the maximum depth with different demineralization time (0.25, 0.5, 0.75, 1, 2, 4, 6, 12, or 24 h). Graph shows the mean and standard deviation of three measurements.

**Fig. 8:**
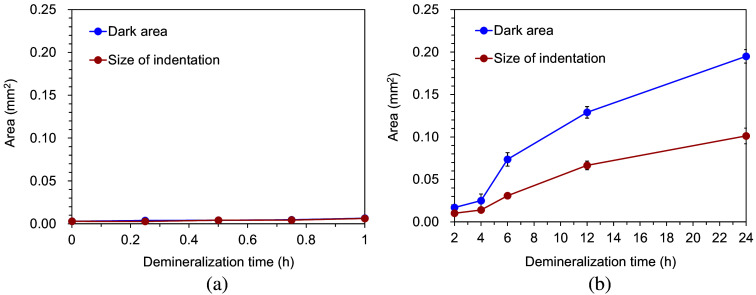
Changes in the dark areas measured by HAMILTOM and indentation size measured by SEM images with different demineralization times. (a) Changes as functions of the demineralization time from 0 to 1 h. (b) Changes as functions of the demineralization time from 2 to 24 h. Graphs show the mean and standard deviation of three measurements.

Figure 7 shows the maximum indentation depth for each demineralization time. The average maximum indentation depth ranged from 2.9 to 3.7  μm. The demineralization time did not significantly affect the results. Figure 8 shows the relationship between the dark areas measured by HAMILTOM and the indentation areas measured by the SEM images for each demineralization time. The difference between the dark areas and the indentation areas increased as the demineralization time increased. For demineralization times of 2 h or less, the dark areas were similar to the indentation areas. However, for demineralization times exceeding 2 h, the dark areas exceeded the indentation areas. The difference became more significant with demineralization time.

**Fig. 9:**
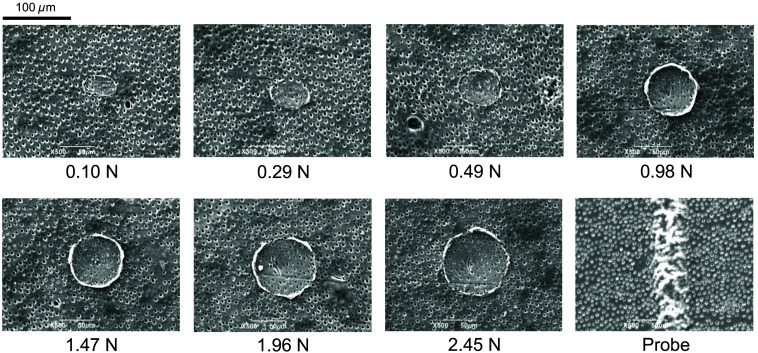
SEM images of indentations on sound dentin samples with various loads of 0.10 to 2.45 N using HAMILTOM or the dental probe based on palpation by the dentist belonging to Osaka Dental University.

**Fig. 10:**
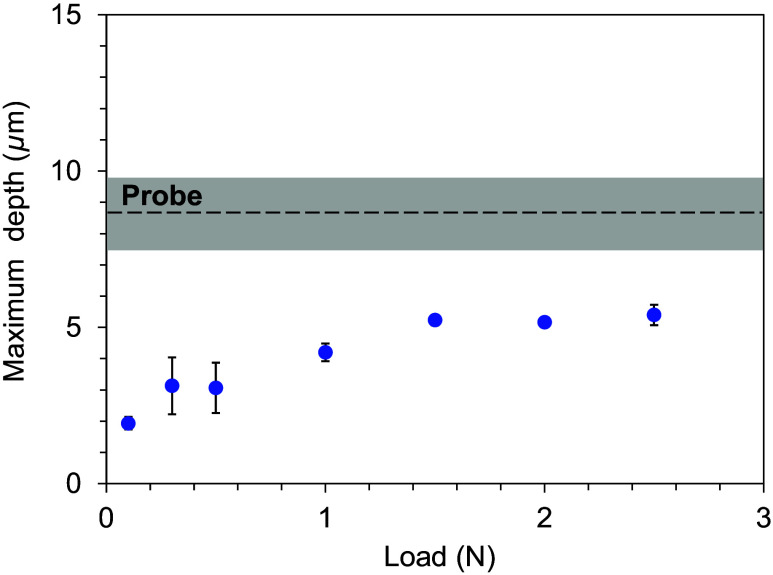
Relationship between the load of HAMILTOM from 10 to 2.45 N and maximum indentation depth on sound dentin samples after hardness measurement by HAMILTOM and a dental probe. Plots and bars show the mean and standard deviation of three measurements by HAMILTOM. Dotted line and the band show the mean and standard deviation of three measurements by the dental probe.

Figure 9 shows SEM images of indentations formed on the sound dentin sample by HAMILTOM with various loads of 0.98 to 2.45 N and those from the dental probe based on palpation by a dentist. The indentation areas increased as the load increased. The dental probe formed a scale-like indentation. Figure 10 shows the maximum indentation depths as functions of load for HAMILTOM and the dental probe. The indentation depths using HAMILTOM increased with load, whereas the indentation depth with the dental probe was 9  μm. The dental probe produced a deeper indentation than that using HAMILTOM for all loads below 2.45 N.

## 4 Discussion

To physically interpret the dentin hardness measured using HAMILTOM, the dark areas and the indentation areas measured by SEM observations for the caries simulated by models with different demineralization times were compared. When the demineralization time is 2 h or less, the dark area is almost the same size as the indentation area. However, the dark area is larger than the indentation area when the demineralization time exceeds 2 h. This difference becomes more pronounced as the demineralization time increases. Thus, the caries simulated models confirmed that the size of the dark area is not the same as the indentation area.

The significant area difference in the dentin samples with a longer demineralization time is attributed to the water taken into the dentin samples during the demineralization reaction. Water has a refractive index of 1.34 at a wavelength of 455 nm,^27^ and the reflectance at the boundary between the indenter and water is so small that the reflected light image is considered to be dark. Caries contains more water than sound dentin.^28^^,^^29^ Pushing an indenter into the dentin sample should exude water from the dentin sample, increasing the dark area in addition to the deformation of the dentin sample. Additionally, the dentinal tubules are closed in the indentation of sound dentin sample formed by HAMILTOM measurements, whereas the tubules are open in the indentation of demineralized dentin samples (Fig. 4). This provides further evidence that water is more likely to seep from demineralized dentin samples due to the load of the indenter than the sound dentin samples.

Elasticity recovery may also contribute to the significant difference between the dark area and indentation area in the caries models for a demineralization time more than 2 h. The deformation gradually rebounds over time. From the SEM image of the indentation by HAMILTOM in Fig. 4, the indentation is contoured when the demineralization time is 1 h or less. However, the contour becomes unclear and the contour ridge increases when the demineralization time is 2 h or more. The modulus of elasticity of the demineralized dentin is 1.6 GPa, suggesting that it is more easily deformed compared to the sound dentin, which has a modulus of elasticity of 19.5 GPa.^30^

The indentation depth measured by the confocal laser microscope (Fig. 5) is very small compared to the depth calculated from the dark area based on the indenter shape used in this study (conical shape with a 90 deg apex angle), suggesting elastic recovery. Therefore, in samples with a long demineralization time, the elastic recovery of the dentin sample after the indenter is removed may also contribute to the significant difference between the dark area and the indentation area. No increase in maximum indentation depth with increasing demineralization time was observed (Fig. 5). It is considered that the dentin sample approached a perfect elastic body and was more likely to recover its elasticity with increasing demineralization time. Elastic recovery is desirable and may further reduce the indentation size of the demineralized dentin over time. From the perspective of invasiveness, the indentation left after the hardness measurement with HAMILTOM should be small and minimally invasive.

Thus, the difference between the dark area measured by HAMILTOM and the actual indentation area may originate from a combination of the water exuded from the dentin sample due to the insertion of the indenter and elastic recovery of the dentin sample after removal of the indenter. To accurately interpret the hardness measured using HAMILTOM, each factor should be investigated independently. Dentin emits green fluorescence upon irradiating teeth with excitation light.^31^ Consequently, we are currently investigating how to isolate each factor using the fluorescence emitted as it may be possible to calculate the percentage of dentin sample deformation in the dark area by fluorescence observations.

Also, the continued improvements should be conducted with respect to the experimental protocols. The Vickers hardness of sound bovine dentin and caries bovine dentin has been reported to 53.5 and 7.7, respectively.^32^ The dentin samples with different hardness were created by changing demineralization time in this study, and the demineralization time of 0 to 24 h created the dentin samples with Vickers harness values from 20 to 52, which were within the range of actual dentin hardness. However, the properties other than hardness associated with demineralization time should also be evaluated. The relationship with the amount of minerals is needed, and an experiment to evaluate the degree of calcification by X-ray micro-computed tomography (micro-CT) is also planned.

Additional research is necessary to discuss the invasiveness of dentin during hardness measurements using HAMILTOM. Comparison of the actual indentation area with the dark area measured by HAMILTOM confirmed that these differ. Since demineralized dentin is often removed after diagnosis, while sound dentin is not, it is critical to consider the invasiveness on sound dentin. The indentation depth by the dental probe with the dentist simulating palpation on sound dentin sample is more than double that by HAMILTOM with a load of 2.45 N (Fig. 8).

Although the probe palpation results in this study are from one dentist and the invasiveness may vary among dentists, the results suggest that HAMILTOM is less invasive than probing during palpation. Here, HAMILTOM defines hardness by measuring the dark area of attenuation at a load of 0.49 N, assuming actual clinical use. However, a dentist operating HAMILTOM may apply a load greater than 0.49 N. Even at a load of 2.45 N, which is significantly higher than the HAMILTOM load of 0.49 N, the indentation depth is less than half that of the dental probe, suggesting that HAMILTOM is less invasive than palpation even for practical use in a clinical setting. Hence, HAMILTOM is minimally invasive. However, other safety risks must be evaluated during actual clinical use.

Although HAMILTOM was found to be minimally invasive, it needs to continue to be improved into a device that is easy to use in clinical practice. Since HAMILTOM is supposed to be used in a handpiece type clinically, the easy hardness measurement is also expected. However, the current prototype is large and needs to be improved for easy clinical hardness measurement. In addition to device miniaturization, the shape of the housing and indenter should be improved to make it easier to apply the indenter to tooth surfaces with reference to dental probes.

Furthermore, it is necessary to verify how much the quantitative evaluation of dentin hardness with HAMILTOM could reduce the diagnostic variability among dentists compared to the conventional qualitative inspection and palpation in actual clinical practice, and a clinical study using HAMILTOM is planned.

For more accurate assessment of the progression of root caries, the easily quantitative evaluation of dentin hardness is required. Especially, the quantitative hardness monitoring could be significant because it is necessary to assess the degree of caries progression in order to prevent the severe root caries. Ultimately, the hardness information measured by HAMILTOM should be able to provide feedback to dentists on the need for treatment.

## 5 Conclusions

Herein dentin hardness measured using HAMILTOM was physically interpreted. Additionally, the dentin hardness measurement mechanism and the invasiveness of HAMILTOM measurements on dentin samples were evaluated to clarify the definition of “hardness” measured using HAMILTOM and evaluate the safety of HAMILTOM for clinical practical application. The results of the comparison of the dark area measured by HAMILTOM and the size of indentation created on the dentin samples observed by SEM images confirmed that the dark areas were not the size of the indentation suggesting the influence of the water exuded by the contact of the indenter and the elastic recovery of the dentin sample after removal of the indenter were included. A comparison of the indentation depths demonstrated that palpation by a dental probe caused a larger indentation depth than HAMILTOM, suggesting that HAMILTOM was less invasive. Hence, HAMILTOM holds promise as a minimally invasive hardness diagnostic method for root caries. In the future, we will continue to investigate the effectiveness and safety of HAMILTOM.

## Data Availability

The data and materials used in this study can be made available upon request via the corresponding author.
